# Tibiofemoral Slip Velocity in Total Knee Arthroplasty is Design-Invariant but Activity-Dependent

**DOI:** 10.1007/s10439-024-03490-4

**Published:** 2024-03-26

**Authors:** Shanyuanye Guan, Raphael Dumas, Marcus G Pandy

**Affiliations:** 1 Department of Mechanical Engineering, University of Melbourne, Parkville, Victoria 3010 Australia; 2 University of Lyon, University Gustave Eiffel, University Claude Bernard Lyon 1, LBMC UMR T_9406, F-69622 Lyon, France

**Keywords:** TKA wear, Archard’s Law, Slip distance, Cross-shear motion, Sliding, Rolling

## Abstract

Tibiofemoral slip velocity is a key contributor to total knee arthroplasty (TKA) component wear, yet few studies have evaluated this quantity *in vivo*. The aim of the present study was to measure and compare tibiofemoral slip velocities in 3 TKA designs for a range of daily activities. Mobile biplane X-ray imaging was used to measure 6-degree-of-freedom tibiofemoral kinematics and the locations of articular contact in 75 patients implanted with a posterior-stabilized, cruciate-retaining, or medial-stabilized design while each patient performed level walking, step up, step down, sit-to-stand, and stand-to-sit. Using these data, tibiofemoral slip velocity was calculated for the duration of each activity for each TKA design. The pattern of tibiofemoral slip velocity was similar for all 3 TKA designs within each activity but markedly different across the 5 activities tested, with the magnitude of peak slip velocity being significantly higher in level walking (range: 158–211 mm/s) than in all other activities (range: 43–75 mm/s). The pattern of tibiofemoral slip velocity in both the medial and lateral compartments closely resembled the pattern of tibiofemoral (knee) flexion angular velocity, with a strong linear relationship observed between slip velocity and flexion angular velocity (*r* = 0.81–0.97). Tibiofemoral slip velocity was invariant to TKA design but was significantly affected by activity type. Our measurements of slip velocity and articular contact locations for a wide range of daily activities may be used as inputs in joint simulator testing protocols and computational models developed to estimate TKA component wear.

**Supplementary Information:**

The online version contains supplementary material available at 10.1007/s10439-024-03490-4.

## Introduction

Total knee arthroplasty (TKA) is a common and effective surgical procedure that corrects joint alignment, relieves pain, and improves mobility and function. Despite the excellent longevity and survivorship of TKA surgery, loosening of the components and periprosthetic osteolysis resulting from polyethylene wear remain frequent causes of long-term TKA failure necessitating revision surgery [1–3]. Wear of the bearing surfaces is dependent on the motion and loading experienced by the implant, which in turn are influenced by the design of the prosthesis and the activities undertaken in daily life.

Archard’s Law for mild wear postulates that the volume of material removed is proportional to the applied load and slip distance [4], where slip distance is given by interfacial slip velocity calculated over a prescribed time interval [5–8]. Although slip velocity is a known contributor to wear [5, 9–12], virtually nothing is known about the effects of TKA component design and activity type on the behavior of this quantity. Direct measurements of knee joint loading have been obtained for various activities of daily living using instrumented prostheses implanted into patients [13], but analogous data for slip velocity are scarce, primarily because of the challenges involved in accurately measuring six-degree-of-freedom (6-DOF) tibiofemoral joint kinematics and the locations of tibiofemoral joint contact *in vivo* [14–17].

Seedhom *et al.* [8] provided a crude estimate of tibiofemoral slip velocity based on gait analysis measurements of knee joint motion obtained for normal walking. Their results showed two prominent peaks in slip velocity, one during early stance and the other in terminal swing. Using video motion capture and a more sophisticated point-cluster marker method, Schwenke *et al.* [10] found tibiofemoral slip velocity to be similar in both compartments of a Miller-Galante TKA prosthesis, with two peaks of similar magnitude occurring in early stance and early swing. Dumas *et al.* [18] also used video motion capture with skin markers to estimate interfacial slip velocity during level walking after total knee replacement. They found that slip velocity peaked during late stance and late swing in both the medial and lateral compartments of the tibiofemoral joint. One of the main limitations of these studies is the error introduced by movement of skin-mounted markers relative to bone [19–21], casting doubt on the accuracy with which marker-based methods may be used to estimate slip velocity *in vivo*.

To our knowledge only two studies have determined tibiofemoral slip velocity more precisely using dynamic X-ray imaging (fluoroscopy). Hamilton *et al.* [5] combined single-plane X-ray fluoroscopy and an elastic foundation contact model to calculate tibiofemoral slip velocity in a posterior-cruciate-ligament-retaining TKA during a stair rise activity. They reported peak slip velocities of approximately 40 mm/s, roughly 4 to 5 times lower than those estimated for walking [10, 18]. More recently, Guan *et al.* [22] measured slip velocity at the counterface in a posterior-stabilized TKA design for level walking and found that slip velocities were similar in the medial and lateral compartments, with peaks of ~ 150 mm/s evident during late stance and late swing. No study has compared tibiofemoral slip velocity across different TKA designs for any activity, including gait, nor has a comparison of slip velocity been performed across a range of daily activities following TKA surgery.

The aim of the present study was to measure and compare tibiofemoral slip velocity across three common TKA designs—posterior stabilized (PS), cruciate retaining (CR), and medial stabilized (MS)—and multiple activities of daily living. Based on a recent finding that tibiofemoral flexion-extension kinematics are similar across these TKA designs [23], we hypothesized that tibiofemoral slip velocity is invariant to TKA design but dependent on activity type.

## Methods

### Participants and TKA Designs

Seventy-five patients were tested 6 months after unilateral TKA surgery (Table 1). Each patient received a randomly assigned PS, CR or MS implant (Medacta International, Switzerland) resulting in three groups with roughly equal numbers of patients (PS: *n* = 23; CR: *n* = 26; MS: *n* = 26). For all TKA designs the chosen post-operative alignment was a 180° hip-knee-ankle angle in the frontal plane with a 0–3° tibial slope in the sagittal plane. The geometry of the tibial component (the tibial tray) was identical in all 3 TKA designs, while the geometry of the femoral component was also identical for the PS and CR designs. The tibial bearings for PS and CR had symmetrical medial and lateral concave plateaus. The MS design was characterized by a spherical medial condyle, a highly conforming medial tibial plateau, and a flat, non-sloping lateral tibial plateau. The anterior cruciate ligament was resected in all three designs, with a cam‐and‐post mechanism incorporated into the PS prosthesis. Geometric features of each TKA design are described in detail by Kour *et al.* [23]. The study was approved by the Human Research Ethics Committees of the University of Melbourne and St. Vincent's Hospital (ID# 1033086).

**Table 1:** Characteristics of the participants in each TKA group

TKA Group	Number of participants			Age	Post-surgery	Height	Weight	BMI	Number of participants in each activity				
	All	Females	Males	(years)	(months)	(cm)	(kg)	(kg/m^2^)	Walk	StepD	StepU	SitD	StandU
PS	23	9	14	66.9 ± 7.4	6.2 ± 1.1	170.3 ± 8.8	90.0 ± 11.6	31.2 ± 4.4	23	23	22	23	23
CR	26	10	16	70.9 ± 7.4	5.9 ± 1.2	169.5 ± 12.5	87.0 ± 14.4	30.5 ± 5.3	25	24	22	26	26
MS	26	14	12	67.3 ± 6.5	6.3 ± 0.9	165.8 ± 10.0	92.0 ± 16.0	33.4 ± 3.9	26	24	20	26	26
All	75	33	42	68.4 ± 7.2	6.2 ± 1.1	168.5 ± 10.7	89.7 ± 14.2	31.7 ± 4.7	74	71	64	75	75

*BMI* body mass index, *PS* posterior-stabilized, *CR* cruciate-retaining, *MS* medial-stabilized, *Walk* level walking, *StepD* step-down, *StepU* step-up, *SitD* stand-to-sit, *StandU* sit-to-stand

### Experimental Protocol

Data were recorded for each participant in a single session at the Biomotion Laboratory, University of Melbourne. The participant wore a lead vest, shorts, and a pair of sandals for the duration of the experiment. Forty-five retroreflective skin markers were attached to the participant’s upper and lower limbs at predetermined locations [24]. Full-body 3D motion, ground reaction force, and biplane X-ray fluoroscopy data were recorded simultaneously for each of the following 5 activities: level walking, step-up, step-down, sit-to-stand and stand-to-sit [23]. The participant practiced each activity prior to data collection.

### Data Collection and Processing

Full-body 3D motion was recorded using a 9-camera video motion capture system (VICON, Oxford, UK) sampling at 120 Hz. Ground reaction forces were measured using two portable strain-gauged force plates (AMTI Accugait, Watertown, MA) sampling at 1080 Hz. Biplane X-ray images (1024 × 1024 pixels, 200 frames/s, 1/200 s exposure time) of the knee were captured using a Mobile Biplane X-ray (MoBiX) imaging system [25, 26].

Walking data were extracted for one complete gait cycle, from heel strike to heel strike of the ipsilateral leg with the TKA implant. Step-up and step-down activities began with ipsilateral heel strike and ended with contralateral heel strike. Sit-to-stand and stand-to-sit activities spanned between minimum and maximum knee flexion. For each participant, data were collected and processed for one trial of each activity. Trials were omitted from analysis if the participant could not perform the activity or if the TKA implant was not captured by the MoBiX imaging system for the entire activity (see Table 1).

**Fig. 1:**
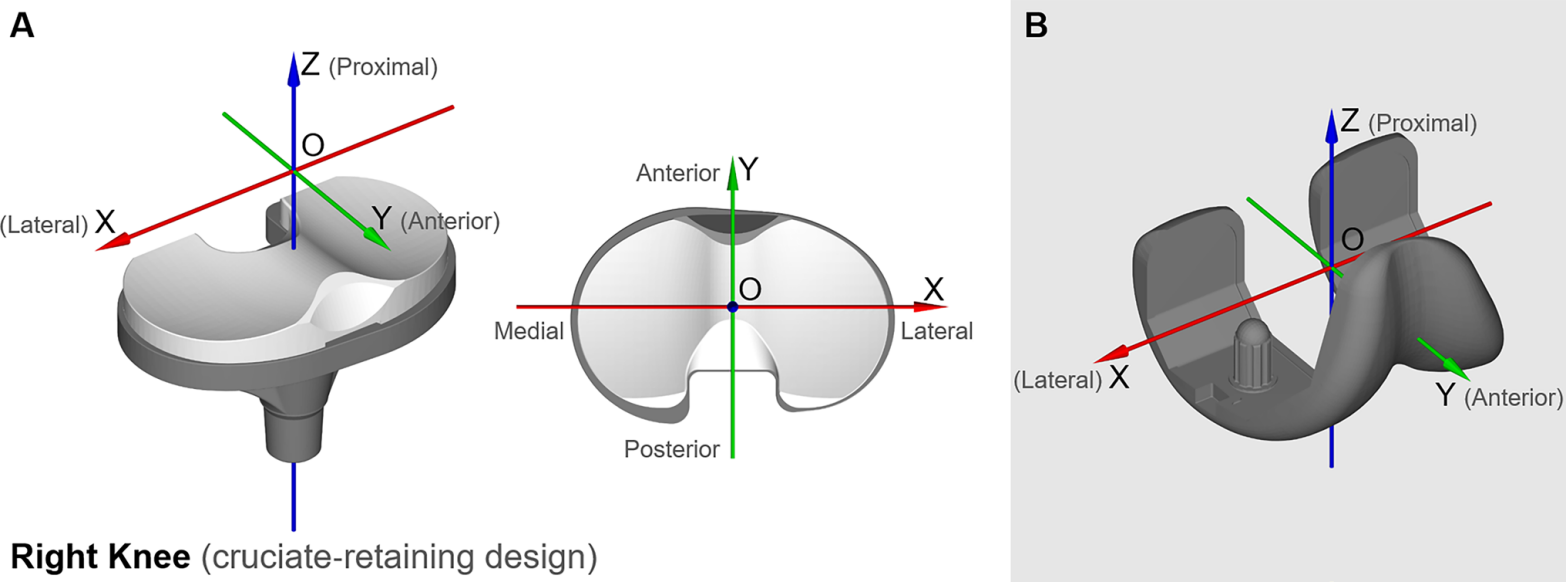
Diagram showing the reference frames used in the present study, which were identical with those defined by Gray *et al.* [25]. The tibial reference frame (panel A) and femoral reference frame (panel B) are illustrated here for a cruciate-retaining (CR) TKA design. **A** For the tibial reference frame, the Z-axis pointed proximally and was coincident with the axis of the tibial stem. The X- and Y-axes were parallel to the transverse plane of the tibial component and pointed anteriorly and to the right, respectively. Slip velocity was calculated and expressed in the tibial reference frame. **B** For the femoral reference frame, the X-axis pointed to the right and was defined as the axis of a cylinder fitted to the posterior and distal surfaces of both femoral condyles. The Z-axis was perpendicular to the transverse flat surface of the femoral component and pointed proximally, whereas the Y-axis pointed anteriorly

Pose-estimation of the femoral and tibial TKA components was performed using the biplane X-ray images together with the 3D geometric models of the TKA implant provided by the manufacturer. Details relating to image processing and pose estimation have been reported previously [26]. 6-DOF tibiofemoral kinematics describing the pose of the tibial component relative to the femoral component were computed and expressed in the anatomical joint coordinate system described by Gray *et al.* [25] (also see Fig. 1). Maximum root-mean-square errors in determining 6-DOF TKA kinematics were 0.33 mm for joint translations and 0.65° for joint rotations [26]. The relative pose between the femoral and tibial components were resampled at 201 equally spaced time points from the beginning to the end of each activity.

The tibiofemoral contact point (i.e., center of the contact region) on the femoral component was approximated as the point on the femoral condyle closest to the tibial bearing in each compartment, and the tibiofemoral slip velocity in each compartment was computed as the velocity of the contact point on the femoral component in the tibial reference frame, thus (see Fig. 1):

1 $${{\varvec{v}}_{{i}}} = \left\{ {\begin{array}{*{20}c} {\frac{\left( {\varvec{p}}_{{{i}}+1}^{{\text{C}}_{{i}}} - {\varvec{p}}_{1}^{{\text{C}}_{{i}}} \right)} {\Delta {{t}}} } & {{{i}}=1} \\ {\frac{\left( {\varvec{p}}_{{{i}}+1}^{{\text{C}}_{{i}}} - {\varvec{p}}_{{{i}}-1}^{{\text{C}}_{{i}}} \right)} {2\Delta {{t}}} } & {1<{{i}}<201} \\ {\frac{\left( {{\varvec{p}}_{201}^{{\text{C}}_{{i}}} - {\varvec{p}}_{{{i}-1}}^{{\text{C}}_{{i}}} } \right)} {\Delta {{t}}} } & {{{i}}=201} \\ \end{array} } \right.$$

where ${\varvec{v}}_{{i}}$ is a vector representing the slip velocity at the ${{i}}^{\text{th}}$ time point, ${\text{C}}_{{i}}$ signifies the contact point on the femoral condyle at the *i*th time point, ${\varvec{p}}_{{{i}}+1}^{{\text{C}}_{{i}}}$ and ${\varvec{p}}_{{{i}}-1}^{{\text{C}}_{{i}}}$ are vectors representing the position of ${\text{C}}_{{i}}$ in the tibial reference frame at time points ${{i}}+1$ and ${{i}}-1$, respectively, $\Delta {{t}}$ is the time interval between two consecutive time points (i.e., 0.5% of the duration of the activity), and a bold face character denotes a vector quantity. The total slip distance ($\varvec{D}$) was calculated in the transverse plane of the tibial component for the duration of each activity using the following equation:

2 $${\varvec{D}} = \Delta {{t}} \mathop \sum \limits_{{{i}}=1}^{201} \left| { {\varvec{v}}_{{{i}}}^{{\text{XY}}} } \right|$$

where $\left|{\varvec{v}}_{{{i}}}^{{\text{XY}}}\right|$ represents the magnitude of the slip velocity ${\varvec{v}}_{{{i}}}$ projected onto the transverse plane of the tibial component at the ${{{i}}}^{{\text{th}}}$ time point (see Fig. 1, Panel A).

### Statistical Analyses

The distribution of peak tibiofemoral slip velocity and total slip distance was tested for normality, and a two-way Analysis of Variance (ANOVA) was first used to determine the effect of TKA design and activity type on peak tibiofemoral slip velocity and total slip distance, with the significance level set to *p* < 0.05. To further investigate the effect of TKA design, for each activity a one‐way ANOVA was used to identify the peak slip velocities and total slip distances that were significantly different (*p* < 0.05) between the 3 TKA designs. Two-tailed, two-sample *t*-tests were then performed on those peak slip velocities and total slip distances to determine whether there was a significant difference between each paired combination of the 3 TKA designs. A conservative threshold of significance was set at *p* < 0.017, obtained by applying a Bonferroni correction for 3 pairwise comparisons with an initial significance threshold of *p* < 0.05. In addition, two-tailed, paired *t*-tests were performed to identify significant differences in peak tibiofemoral slip velocity and total slip distance between the medial and lateral compartments of the tibiofemoral joint for each TKA design and each activity, with a threshold of *p* < 0.05. Finally, a correlation analysis was conducted to determine the relationship between tibiofemoral slip velocity and tibiofemoral (knee) flexion angular velocity.

## Results

**Fig. 2:**
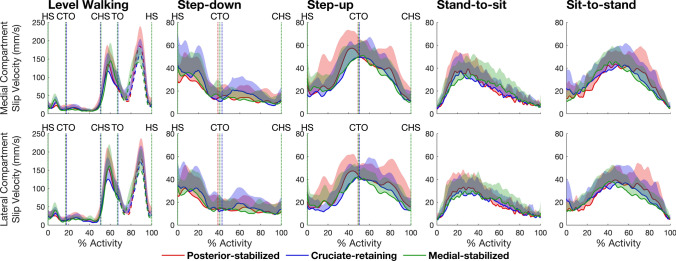
Magnitude of the resultant slip velocities measured in the medial compartment (top row) and the lateral compartment (bottom row) of the tibiofemoral joint for the 3 TKA designs and 5 activities tested. Data were measured at 201 equally spaced time points from the beginning to the end of each activity. The durations of level walking, step-down, step-up, stand-to-sit and sit-to-stand were 1.33 ± 0.16, 0.73 ± 0.33, 0.98 ± 0.18, 2.20 ± 0.75, 1.58 ± 0.38 seconds, respectively. The lines in each plot represent the mean slip velocity measured for each group and the shaded regions represent 1 standard deviation from the mean. For level walking, the solid and dashed lines represent the stance and swing phases, respectively. The vertical dotted lines mark key events during each activity: *HS* heel‐strike, *CTO* contralateral toe‐off, *CHS* contralateral heel‐strike, *TO* toe‐off

Peak tibiofemoral slip velocity was significantly higher for level walking than for all other activities (Fig. 2). Slip velocity peaked thrice during level walking: first in early stance shortly after heel-strike, and then during late stance and mid-swing. Mean peak slip velocity in both the medial and lateral compartments was highest during swing (range: 153–211 mm/s), slightly lower during late stance (range: 149–187 mm/s), and considerably lower during early stance (range: 36–45 mm/s) for all 3 TKA designs (Table 2, Panel A). Mean peak slip velocities for all other activities ranged from 43 to 75 mm/s in both the medial and lateral compartments for all 3 TKA designs. For all TKA designs and all activities, except level walking, peak slip velocity was higher (by 7–19%) in the medial compartment than the lateral compartment. In level walking, peak slip velocity was higher (by 5–21%) in the lateral compartment than the medial compartment across all 3 TKA designs (Table 3, Panel A).

**Table 2:** Peak slip velocities (Panel A) and total slip distances (Panel B) measured at the medial and lateral compartments of the tibiofemoral joint for the 5 activities tested. Total slip distance was computed relative to the transverse plane of the tibial bearing over the duration of each activity.

		Mean ± Standard Deviation			ANOVA	PS vs CR			PS vs MS			CR vs MS		
		PS	CR	MS	*P*-value	Diff.	95% CI	*P*-value	Diff.	95% CI	*P*-value	Diff.	95% CI	*P*-value
***(A) Peak slip velocity (mm/s)***														
Medial	Walk Peak 1	35.9 ± 15.2	39.9 ± 14.3	41.1 ± 17.3	0.487	− 4.0	− 12.6 to 4.6	0.351	− 5.2	− 14.6 to 4.2	0.271	− 1.2	− 10.1 to 7.7	0.789
	Walk Peak 2	173.1 ± 38.2	149.2 ± 37.8	158.0 ± 39.5	0.102	24.0	1.9 to 46.0	0.034	15.1	− 7.3 to 37.5	0.181	− 8.8	− 30.6 to 12.9	0.418
	Walk Peak 3	211.1 ± 48.3	180.1 ± 37.8	152.7 ± 34.8	** < 0.001**^**a**^	31.0	5.9 to 56.1	0.017*	58.4	34.4 to 82.4	< 0.001*	27.4	7.0 to 47.9	0.010*
	Step-down	49.0 ± 21.8	62.6 ± 31.1	57.3 ± 23.3	0.198	− 13.6	− 29.4 to 2.2	0.090	− 8.3	− 21.6 to 5.0	0.214	5.3	− 10.7 to 21.3	0.506
	Step-up	75.4 ± 19.5	65.7 ± 13.3	64.1 ± 13.7	**0.048**^**a**^	9.6	− 0.5 to 19.8	0.063	11.3	0.7 to 21.9	0.038	1.7	− 6.8 to 10.1	0.694
	Stand-to-sit	56.6 ± 12.1	49.6 ± 13.8	52.2 ± 12.4	0.166	7.0	− 0.5 to 14.5	0.067	4.4	− 2.7 to 11.5	0.217	− 2.6	− 9.9 to 4.7	0.478
	Sit-to-stand	58.8 ± 15.1	61.1 ± 14.1	56.6 ± 10.1	0.473	− 2.2	− 10.6 to 6.1	0.594	2.3	− 5.0 to 9.6	0.537	4.5	− 2.3 to 11.3	0.191
Lateral	Walk Peak 1	40.7 ± 15.4	44.1 ± 17.5	45.4 ± 20.6	0.655	− 3.4	− 13.0 to 6.2	0.484	− 4.7	− 15.2 to 5.9	0.380	− 1.3	− 12.1 to 9.5	0.811
	Walk Peak 2	187.3 ± 43.8	159.2 ± 44.9	175.0 ± 50.7	0.119	28.1	2.3 to 53.9	0.033	12.3	− 15.1 to 39.7	0.372	− 15.8	− 42.8 to 11.2	0.244
	Walk Peak 3	198.3 ± 51.8	186.6 ± 46.8	166.3 ± 47.1	0.070	11.7	− 17.0 to 40.3	0.417	32.0	3.6 to 60.4	0.028	20.3	− 6.1 to 46.8	0.128
	Step-down	44.8 ± 19.1	55.0 ± 20.3	53.9 ± 18.6	0.148	− 10.3	− 21.8 to 1.3	0.081	− 9.1	− 20.2 to 2.0	0.104	1.1	− 10.2 to 12.5	0.841
	Step-up	66.4 ± 16.6	57.2 ± 13.1	54.8 ± 13.7	**0.030**^**a**^	9.1	0.0 to 18.2	0.049	11.5	2.0 to 21.1	0.019	2.4	− 6.0 to 10.8	0.565
	Stand-to-sit	50.9 ± 9.8	42.9 ± 12.2	50.0 ± 14.3	**0.049**^**a**^	7.9	1.5 to 14.3	0.016*	0.8	− 6.3 to 8.0	0.817	− 7.1	− 14.5 to 0.3	0.060
	Sit-to-stand	54.2 ± 13.0	54.8 ± 12.4	50.1 ± 11.9	0.343	− 0.6	− 7.9 to 6.7	0.878	4.1	− 3.0 to 11.3	0.253	4.7	− 2.1 to 11.5	0.172
***(B) Total slip distance (mm)***														
Medial	Level walking	66.1 ± 9.8	62.6 ± 10.9	62.0 ± 9.7	0.335	3.5	− 2.5 to 9.5	0.249	4.0	− 1.6 to 9.6	0.155	0.5	− 5.3 to 6.3	0.854
	Step-down	10.9 ± 4.5	13.5 ± 5.7	12.1 ± 4.3	0.185	− 2.6	− 5.7 to 0.4	0.086	− 1.2	− 3.8 to 1.3	0.334	1.4	− 1.5 to 4.3	0.344
	Step-up	30.9 ± 6.6	30.0 ± 5.9	29.9 ± 5.4	0.842	0.9	− 2.9 to 4.7	0.647	1.0	− 2.8 to 4.8	0.603	0.1	− 3.4 to 3.7	0.949
	Stand-to-sit	42.3 ± 6.1	41.5 ± 6.4	40.5 ± 6.0	0.588	0.8	− 2.9 to 4.4	0.676	1.8	− 1.7 to 5.3	0.300	1.1	− 2.4 to 4.5	0.542
	Sit-to-stand	42.5 ± 5.8	41.7 ± 5.6	39.9 ± 5.4	0.281	0.8	− 2.5 to 4.1	0.640	2.5	− 0.7 to 5.7	0.126	1.7	− 1.3 to 4.8	0.264
Lateral	Level walking	70.1 ± 12.8	65.5 ± 14.6	65.0 ± 13.2	0.375	4.5	− 3.5 to 12.5	0.260	5.0	− 2.5 to 12.5	0.184	0.5	− 7.3 to 8.3	0.900
	Step-down	10.4 ± 4.3	12.3 ± 4.5	11.0 ± 3.4	0.283	− 1.9	− 4.4 to 0.7	0.155	− 0.6	− 2.8 to 1.7	0.623	1.3	− 1.0 to 3.6	0.263
	Step-up	27.3 ± 6.4	25.2 ± 5.6	25.6 ± 5.0	0.436	2.1	− 1.6 to 5.8	0.254	1.7	− 1.9 to 5.3	0.339	− 0.4	− 3.7 to 2.9	0.822
	Stand-to-sit	39.3 ± 6.5	38.2 ± 7.4	36.7 ± 5.9	0.379	1.2	− 2.8 to 5.2	0.558	2.7	− 0.9 to 6.2	0.142	1.5	− 2.3 to 5.2	0.431
	Sit-to-stand	38.7 ± 6.0	36.9 ± 6.0	33.9 ± 5.5	**0.019**^**a**^	1.7	− 1.7 to 5.2	0.313	4.7	1.4 to 8.1	0.006*	3.0	− 0.2 to 6.2	0.067

Peaks 1, 2, and 3 are the peak slip velocities during level walking in regions of HS-CTO, CHS-TO, and TO-HS, respectively.

^**a**^Bold type indicates P < 0.05 for ANOVA

*Bold type indicates P < 0.017 for *t* tests

*PS* posterior-stabilized, *CR* cruciate-retaining, *MS* medial-stabilized, *Diff*. difference, *CI* confidence interval, *HS* heel‐strike, *CTO* contralateral toe‐off, *CHS* contralateral heel‐strike, *TO* toe‐off, *Walk* level walking

**Table 3:** Comparisons of peak slip velocity and total slip distance between the medial and lateral compartments of the tibiofemoral joint. A positive difference means the value associated with the medial compartment was higher than that associated with the lateral compartment.

	**Posterior-stabilized**			**Cruciate-retaining**			**Medial-stabilized**		
	Diff.	95% CI	*P*-value	Diff.	95% CI	P-value	Diff.	95% CI	*P*-value
***(A) Peak slip velocity (mm/s)***									
Walk Peak 1	− 4.8	− 10.2 to 0.5	0.073	− 4.2	− 8.9 to 0.6	0.081	− 4.3	− 10.3 to 1.7	0.154
Walk Peak 2	− 14.2	− 21.9 to − 6.6	0.001*	− 10.1	− 19.3 to − 0.8	0.034*	− 17.1	− 28.9 to − 5.2	0.007*
Walk Peak 3	12.8	− 0.6 to 26.2	0.061	− 6.5	− 23.4 to 10.3	0.431	− 13.6	− 28.9 to 1.6	0.078
Step-down	4.2	− 1.1 to 9.6	0.116	7.6	− 1.2 to 16.4	0.088	3.4	− 2.5 to 9.3	0.244
Step-up	9.0	3.5 to 14.5	0.003*	8.5	3.9 to 13.1	< 0.001*	9.2	5.0 to 13.5	< 0.001*
Stand-to-sit	5.7	2.1 to 9.4	0.004*	6.7	3.7 to 9.6	< 0.001*	2.2	− 1.7 to 6.0	0.259
Sit-to-stand	4.6	− 0.3 to 9.5	0.066	6.3	3.0 to 9.6	< 0.001*	6.5	4.1 to 8.9	< 0.001*
***(B) Total slip distance (mm)***									
Level walking	− 4.0	− 6.7 to − 1.4	0.005*	− 3.0	− 6.4 to 0.4	0.085	− 3.0	− 6.6 to 0.5	0.094
Step-down	0.5	− 0.4 to 1.3	0.261	1.2	0.0 to 2.4	0.043	1.2	0.3 to 2.0	0.009
Step-up	3.6	2.7 to 4.5	< 0.001*	4.8	3.5 to 6.1	< 0.001*	4.3	3.0 to 5.7	< 0.001*
Stand-to-sit	2.9	2.0 to 3.9	< 0.001*	3.4	2.1 to 4.6	< 0.001*	3.8	2.5 to 5.1	< 0.001*
Sit-to-stand	3.8	2.7 to 4.8	< 0.001*	4.7	3.7 to 5.8	< 0.001*	6.0	4.9 to 7.1	< 0.001*

*Bold type indicates P < 0.05 for paired *t* tests.

*Diff*. difference, *CI* confidence interval, *Walk* level walking

**Fig. 3:**
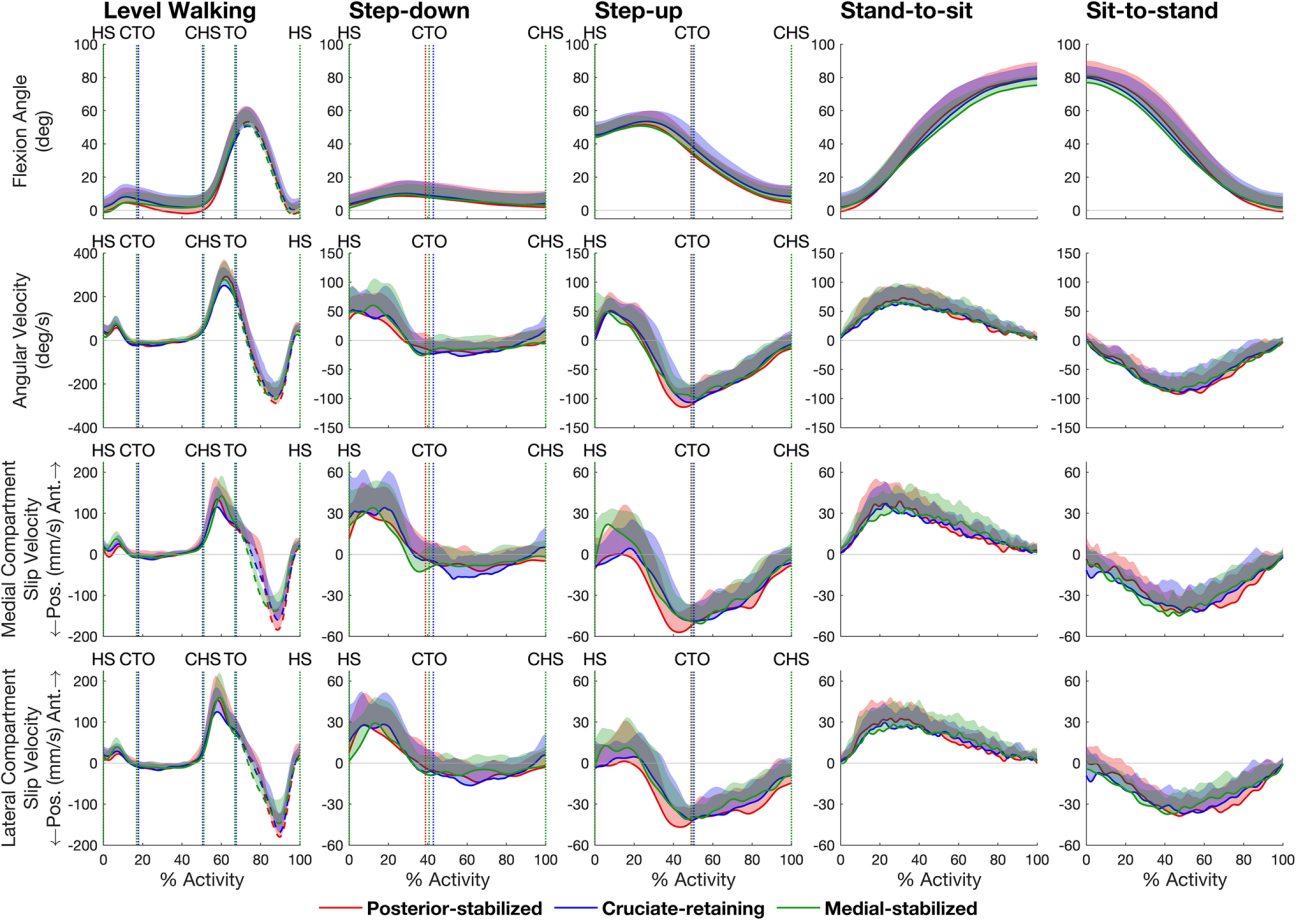
Tibiofemoral flexion angle (top row), tibiofemoral flexion angular velocity (second row), and slip velocities measured in the anterior-posterior direction in the medial and lateral compartments (bottom two rows) of the tibiofemoral joint for the 3 TKA designs and 5 activities tested. Data were measured at 201 equally spaced time points from the beginning to the end of each activity. The lines in each plot represent the mean value of each quantity measured for each group and the shaded regions represent 1 standard deviation from the mean. For level walking, the solid and dashed lines represent stance and swing, respectively. Vertical dotted lines mark key events during each activity: *HS* heel‐strike, *CTO* contralateral toe‐off, *CHS* contralateral heel‐strike, *TO* toe‐off, *Pos*. posterior, *Ant*. anterior. (Corresponding slip velocities in the medial-lateral and inferior-superior directions are given in Supplementary Figure S1)

For all TKA designs and all activities, the patterns of medial and lateral tibiofemoral slip velocity closely resembled the pattern of tibiofemoral flexion angular velocity, with peak slip velocities occurring at roughly the same time instants as the peaks in tibiofemoral flexion angular velocity (Fig. 3, compare second, third and bottom rows). Knee flexion resulted in anteriorly directed velocities of the contact point on the femur relative to the tibia, whereas knee extension coincided with posteriorly directed velocities of the contact point for all TKA designs and all activities (Fig. 3, first, third and bottom rows).

**Fig. 4:**
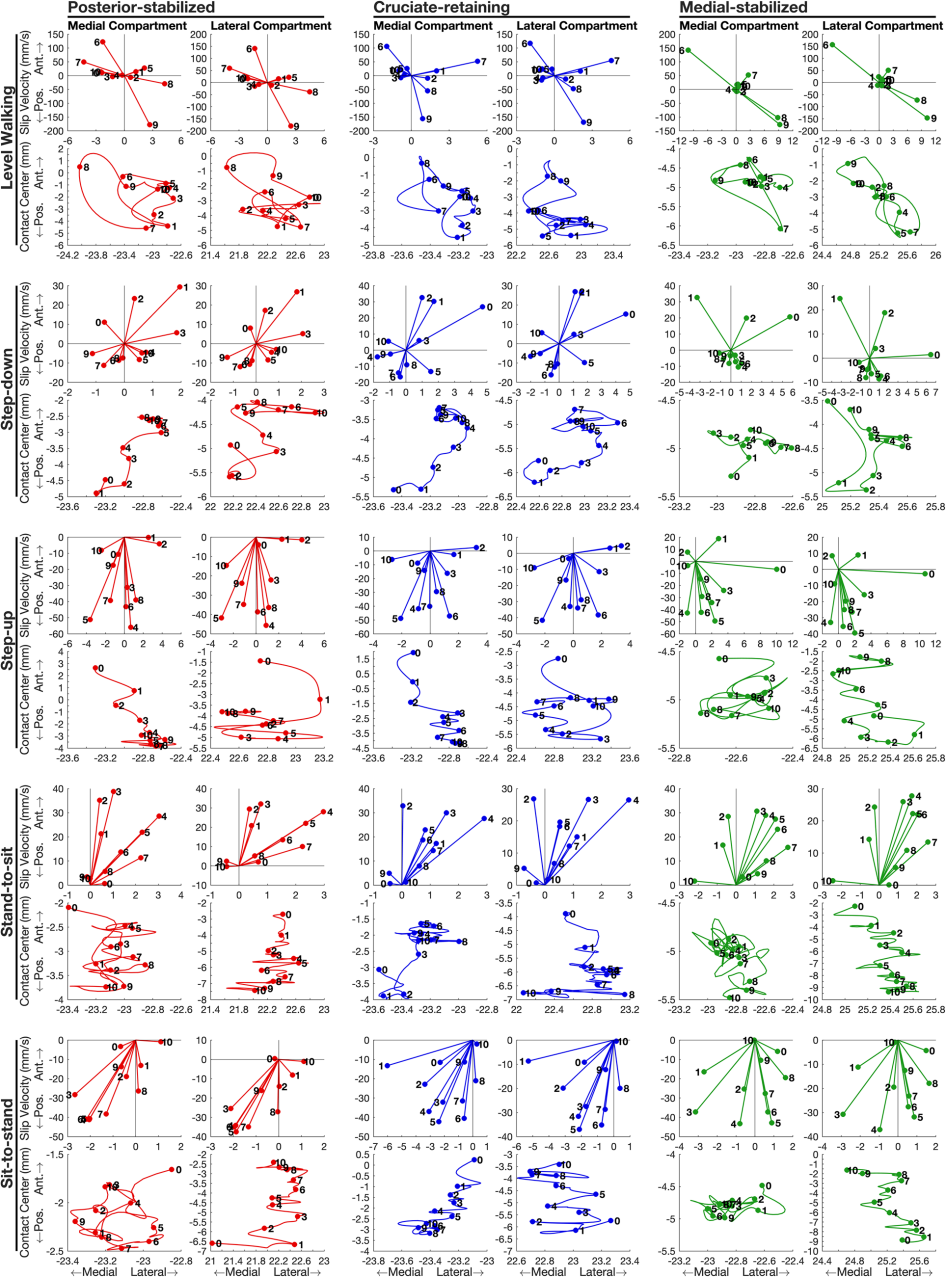
Resultant slip velocity and the path of the tibiofemoral contact center measured for the medial and lateral compartments of the tibiofemoral joint. Data were averaged across all participants in each TKA group and projected onto the transverse plane of the tibial bearing for each of the 3 TKA designs (posterior-stabilized, columns 1–2; cruciate-retaining, columns 3–4; medial-stabilized, columns 5–6) and each of the 5 activities (Level walking, rows 1–2; Step down, rows 3–4; Step up, rows 5–6; Stand-to-sit, rows 7–8; sit-to-stand, rows 9–10). For each activity, the top row shows the resultant slip velocity, and the bottom row shows the trajectory of the contact center projected onto the tibial bearing surface. The dots represent either slip velocity or the location of the contact point plotted at time increments of 10% during each activity. The number beside each dot signifies the time sequence of the activity; that is, 0, 1, …, and 10 designate the slip velocity or the location of the contact center at 0%, 10%, …, and 100% of the activity, respectively. The path of each contact center was obtained at 201 equally spaced time points from the beginning to the end of each activity. Note that the scale in the anterior-posterior direction is approximately 40 times greater than the scale in the medial-lateral direction. *Pos*. posterior, *Ant*. anterior

Peak slip velocities in the anterior-posterior direction were more than 1 order of magnitude greater than those in the medial-lateral direction for both the medial and lateral compartments across all TKA designs and all activities (Fig. 4, top row for each activity; see also Fig. S1 of Supplemental Material). The magnitude of slip velocity in the superior-inferior direction (Fig. S1) was comparable to if not smaller than that in the medial-lateral direction. Peak-to-peak anterior-posterior displacements of the tibiofemoral contact centers in the medial and lateral compartments of PS and CR were similar for all activities (Fig. 4, bottom row for each activity). For MS, peak-to-peak anterior-posterior displacement of the contact center in the medial compartment was significantly smaller than that in the lateral compartment for all activities (Fig. 4, Medial-stabilized, bottom row for each activity; see also Fig. S2, bottom row, in Supplemental Material). The contact centers in the medial and lateral compartments of all 3 TKA designs underwent multi-directional motions during each activity, with multiple crossings of the motion path traced on the tibial bearing over the course of each cycle.

Two-way ANOVA revealed that activity type significantly influenced both peak slip velocity and total slip distance (*p* < 0.001) (Table 4). Additionally, TKA design emerged as a factor affecting peak slip velocity in the medial compartment (*p* = 0.005) and total slip distance in the lateral compartment (*p* = 0.028). Notably, the much higher F-values associated with activity type compared to those for TKA design indicate that peak slip velocity and total slip distance were more profoundly affected by activity type than TKA design. Specifically, activity type accounted for 81.5% of the total variance in peak slip velocity in the medial compartment, compared to a meagre 0.6% explained by TKA design. Similarly, for total slip velocity in the lateral compartment, activity type explained 84.3% of the total variance while TKA design contributed just 0.3%.

**Table 4:** Two-way ANOVA showing effects of TKA design and activity type on peak slip velocity and total slip distance

		Medial Compartment				Lateral Compartment			
Source	DOF	Sum of Sq.	Mean Sq.	*F*-value	*P*-value	Sum of Sq.	Mean Sq.	*F*-value	*P*-value
***(A) Peak slip velocity (mm/s)***									
TKA design	2	6730.5	3365.2	5.4	**0.005**^**a**^	1989.1	994.5	1.5	0.236
Activity	4	996740.3	249185.1	398.5	** < 0.001**^**a**^	1223705.6	305926.4	446.6	** < 0.001**^**a**^
Error	352	220134.0	625.4			241132.6	685.0		
Total	358	1222284.4				1466459.1			
***(B) Total slip distance (mm)***									
TKA design	2	164.5	82.2	1.8	0.173	455.0	227.5	3.6	**0.028**^**a**^
Activity	4	100763.6	25190.9	540.2	** < 0.001**^**a**^	120435.0	30108.7	477.6	** < 0.001**^**a**^
Error	352	16413.3	46.6			22190.4	63.0		
Total	358	117232.0				142858.8			

^a^Bold type indicates *P* < 0.05 for ANOVA

*DOF* degrees of freedom, *Sq*. squares

One-way ANOVA performed for each individual activity revealed that peak tibiofemoral slip velocity was invariant to TKA design across all activities, except for the following four instances related to level walking, step-up, and stand-to-sit (Table 2, Panel A). There was a significant effect of TKA design on slip velocity for the swing phase of level walking (*p* < 0.001) (Table 2, Panel A, Walk Peak 3). Peak slip velocity was higher for PS compared to CR (by 31.0 mm/s, *p* < 0.017), for PS compared to MS (by 58.4 mm/s, *p* < 0.001), and for CR compared to MS (by 27.4 mm/s, *p* = 0.010), but only in the medial compartment of each TKA design. There was also a significant effect of TKA design on slip velocity for stand-to-sit (*p* = 0.049), where peak slip velocity in the lateral compartment was higher for PS compared to CR (by 7.9 mm/s, *p* = 0.016) (Table 2, Panel A, Stand-to-sit). A significant effect of TKA design on slip velocity was also found for step-up in both the medial and lateral compartments (*p* = 0.048 and p = 0.030, respectively), but differences in peak slip velocity between each paired combination of the PS, CR, and MS designs were not significant (Table 2, Panel A, Step-up).

Total tibiofemoral slip distance was invariant to TKA design for all activities except sit-to-stand, where total slip distance in the lateral compartment of PS was higher than that for MS (by 4.7 mm, *p* = 0.006) (Table 2, Panel B). Total slip distance was highest for level walking and lowest for step down. For all TKA designs and all activities, except level walking, total slip distance was higher (by 6–20%) in the medial compartment than the lateral compartment. In level walking, total slip distance was higher (by 5–6%) in the lateral compartment than the medial compartment across all TKA designs (Table 3, Panel B).

**Fig. 5:**
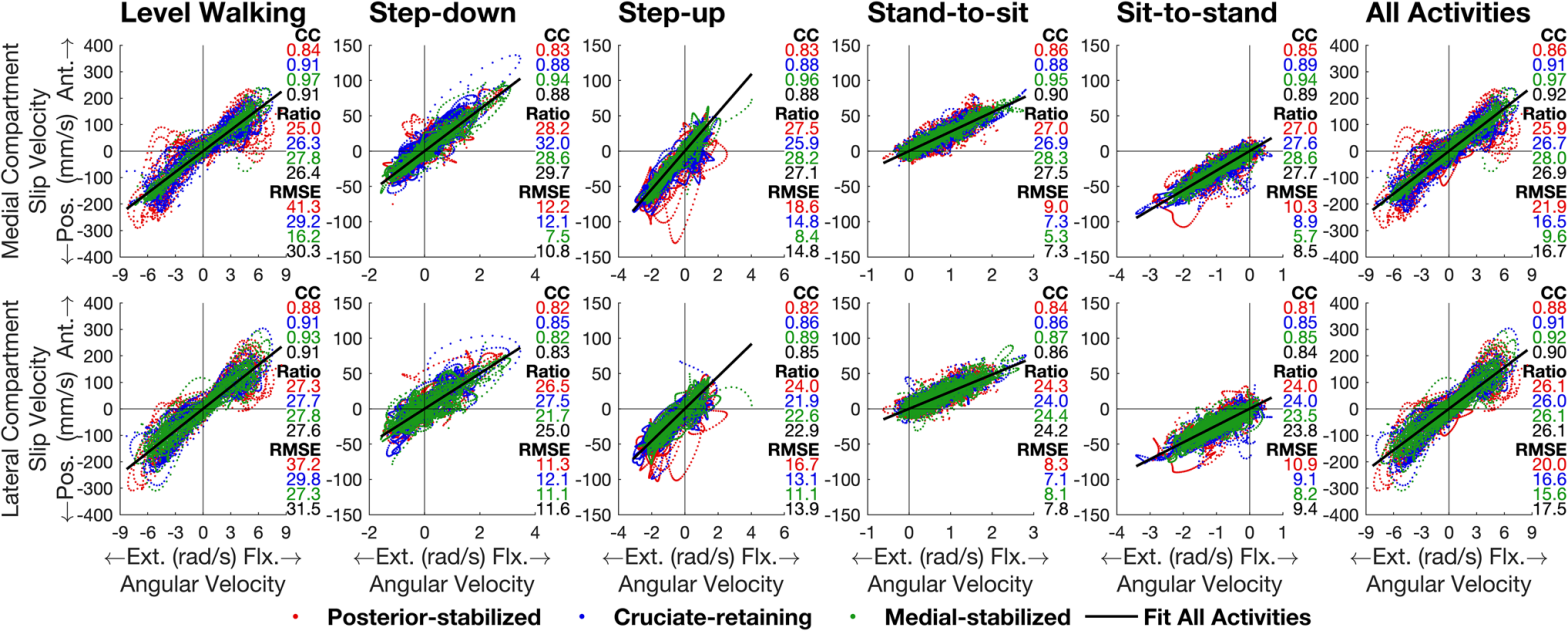
Magnitude of the anterior-posterior component of the slip velocity measured in the medial compartment (top row) and the lateral compartment (bottom row) of the tibiofemoral joint plotted against the magnitude of the tibiofemoral (knee) flexion angular velocity for the 3 TKA designs and 5 activities tested. Pearson correlation coefficients (“CC”), ratio of slip velocity to angular velocity (“Ratio” in mm), and Root Mean Square Error (“RMSE” in mm/s) are given on the right-hand side of each panel. Each red, blue, and green dot signifies the slip velocity and its corresponding angular velocity for the posterior-stabilized, cruciate-retaining, and medial-stabilized TKA designs, respectively. The dots in the first five columns represent the measured values of slip velocity and knee joint angular velocity for each of the 5 activities plotted for all individual patients in each TKA group, whereas the dots in the last column (All Activities) represent the measured values of slip velocity and knee joint angular velocity for all 5 activities pooled together. A line passing through the origin was fitted to the dots of each color in each panel (not shown). The black line shown in each panel was fitted to all the colored dots pooled in each panel. The slope of each line of best fit is given as a “Ratio” on the right-hand side of each panel. *Pos*. posterior, *Ant*. anterior, *Ext*. extension, *Flx*. flexion. See text for the equations specifying the lines of best fit shown in the far-right panels

Slip velocities in the medial and lateral compartments of the tibiofemoral joint were highly correlated with and linearly related to tibiofemoral flexion angular velocity for all TKA designs and all activities (Pearson correlation coefficient *r* = 0.86–0.97) (Fig. 5, All Activities). The slope of the linear relationship between slip velocity and flexion angular velocity was approximately equal to the radius of the femoral condyles (PS: 26.0 ± 1.0 mm; CR: 25.7 ± 1.4 mm; MS: 29.2 ± 1.8 mm; All: 27.0 ± 2.2 mm; see also Table S1 of Supplemental Material) for both the medial and lateral compartments across all TKA designs and all activities (Fig. 5, All Activities). The radii of the medial and lateral femoral condyles were equal for each TKA design. The equations relating slip velocity and flexion angular velocity for the medial and lateral compartments across all TKA designs and all activities are given by:

3 $${{V}}_{{{\text{Med}}}} = 26.9{{\omega}}$$

4 $${{V}}_{{{\text{Lat}}}} = 26.1{{\omega}}$$

where ${{{V}}}_{{\text{Med}}}$ and ${{{V}}}_{{\text{Lat}}}$ are respectively the tibiofemoral slip velocities for the medial and lateral compartments (mm/s), and ${\omega}$ is the tibiofemoral flexion angular velocity (rad/s).

## Discussion

We found that the pattern of tibiofemoral slip velocity was similar for all 3 TKA designs within each activity but markedly different across the 5 activities tested (Fig. 2). For all TKA designs, peak slip velocity was significantly higher in level walking (range: 158–211 mm/s) than in all other activities (range: 43–75 mm/s) (Table 2, Panel A). Total slip distance was also similar for all TKA designs within each activity but differed significantly across activities (Table 2, Panel B). The pattern of tibiofemoral slip velocity in both the medial and lateral compartments closely resembled the pattern of tibiofemoral (knee) flexion angular velocity (Fig. 3), with a strong linear relationship observed between slip velocity and flexion angular velocity (*r* = 0.81–0.97) (Fig. 5). Two-way and one-way ANOVA showed that peak slip velocity was invariant to TKA design within each activity but there was a significant effect of activity type on peak slip velocity, thus our main hypothesis was supported.

**Fig. 6:**
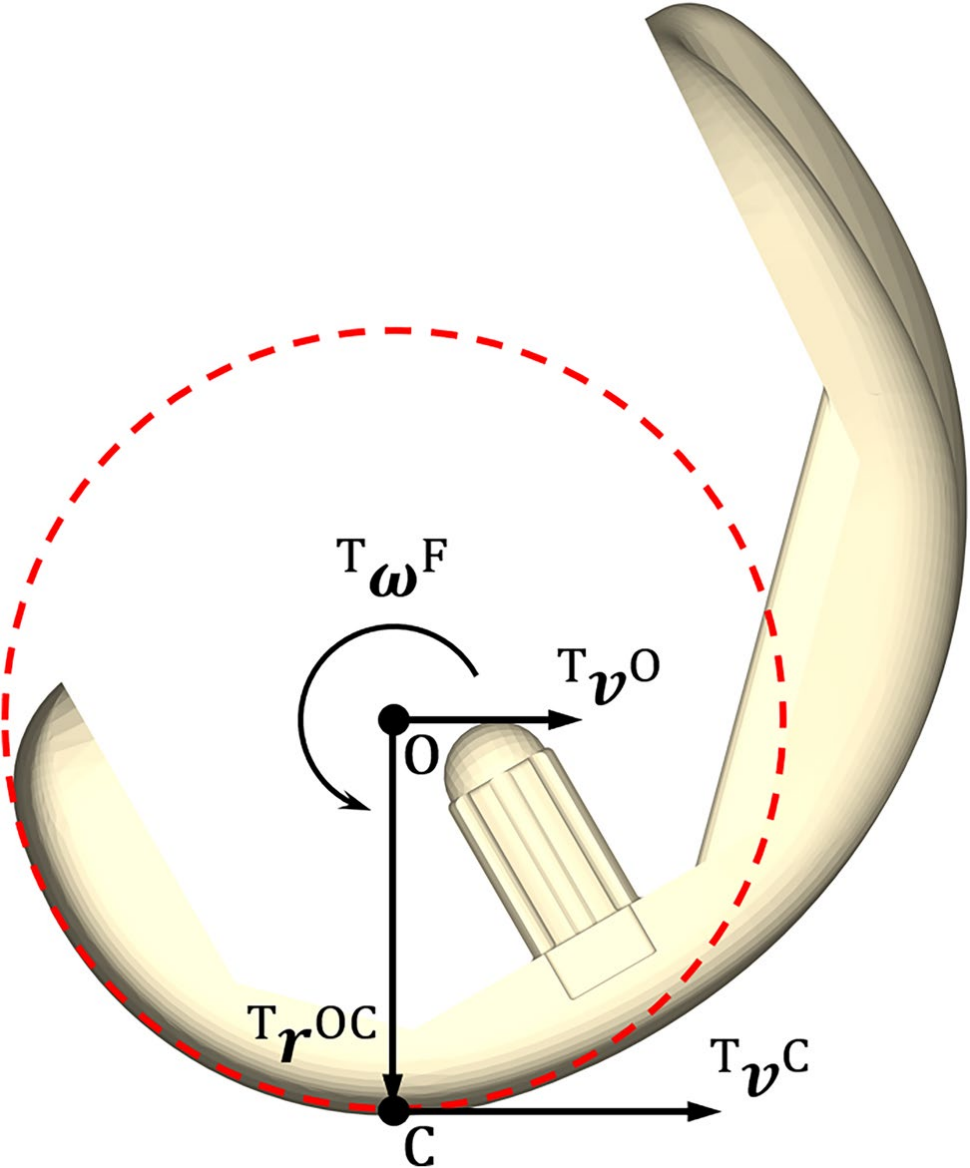
Diagram illustrating the relationship between the velocity of the tibiofemoral contact center (point C) and the velocity of the center of the femoral condyle (point O). Both points C and O are points fixed on the femoral component. All the linear and angular velocities are expressed in the tibial reference frame (T) fixed on the tibial bearing (see Fig. 1). $^{{\text{T}}} {{\varvec{v}}}^{{\text{C}}}$ is the linear velocity of the tibiofemoral contact center, point C, in T; $^{{\text{T}}} {{\varvec{v}}}^{{\text{O}}}$ is the linear velocity of the center of the femoral condyle, point O, in T; $^{{\text{T}}} {{\varvec{\omega}}}^{{\text{F}}}$ is the angular velocity of the femur, body F, in T; and $^{{\text{T}}}{\varvec{r}}^{{\text{OC}}}$ is the position vector directed from point O to point C, in T. The cross-product $^{{\text{T}}} {{\varvec{\omega}}}^{{\text{F}}} \times ^{{\text{T}}}{\varvec{r}}^{{\text{OC}}}$ represents the velocity of the contact center, point C, relative to the center of the femoral condyle, point O

**Fig. 7:**
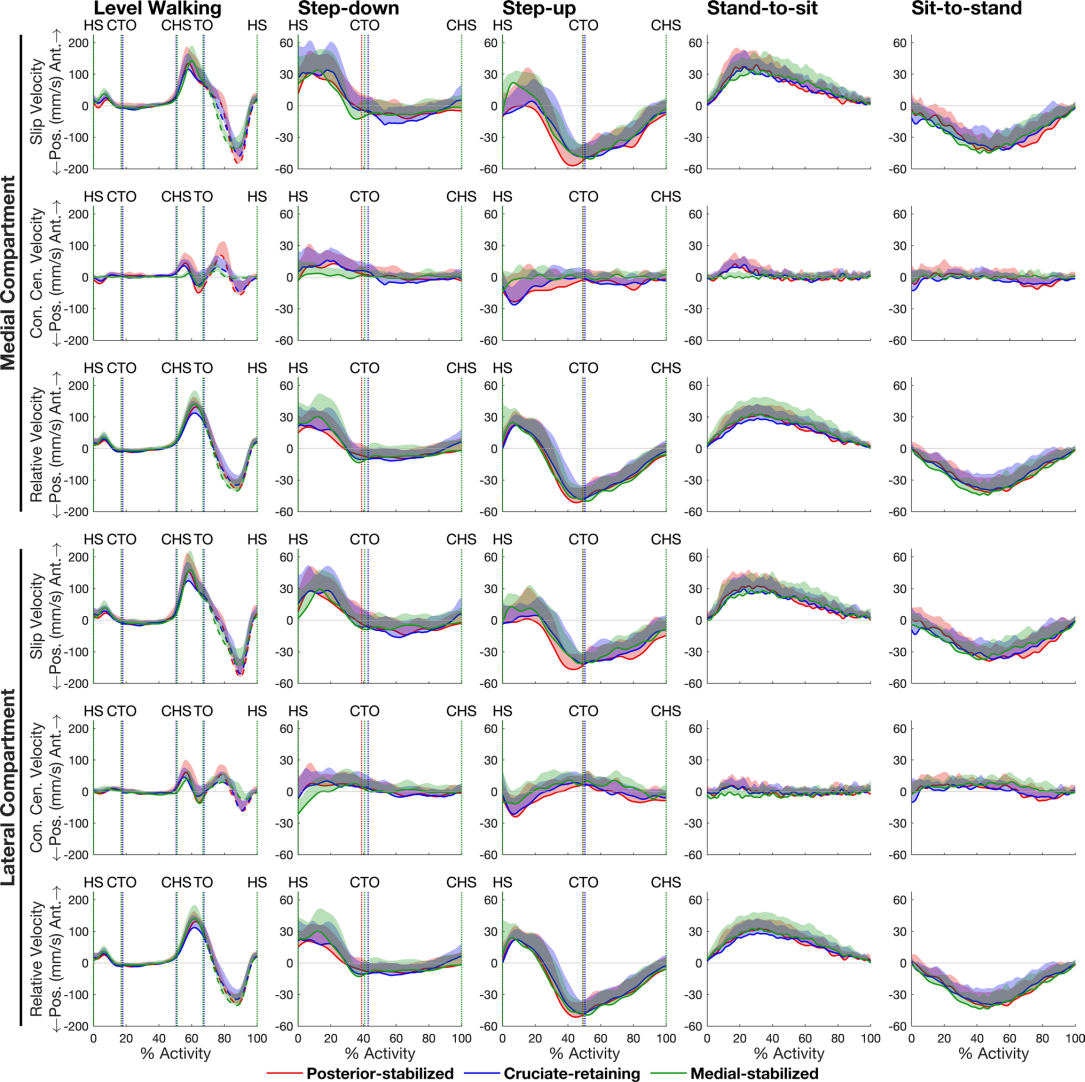
Time histories of the anterior-posterior component of the slip velocity ($^{{\text{T}}} {{\varvec{v}}}^{{\text{C}}}$ in Fig. 6) (rows 1 and 4), linear velocity of the center of the femoral condyle ($^{{\text{T}}} {{\varvec{v}}}^{{\text{O}}}$ in Fig. 6) (rows 2 and 5), and the velocity of the tibiofemoral contact center relative to the center of the femoral condyle ($^{{\text{T}}} {{\varvec{\omega}}}^{{\text{F}}} \times ^{{\text{T}}}{\varvec{r}}^{{\text{OC}}}$ in Fig. 6) (rows 3 and 6) for the medial compartment (top 3 rows) and the lateral compartment (bottom 3 rows) of the tibiofemoral joint for the 3 TKA designs and 5 activities tested. The lines in each plot represent the mean value of each quantity measured for each group while the shaded regions represent 1 standard deviation from the mean. The magnitude of the velocity of the contact center, point C, relative to the center of the femoral condyle, point O, in Fig. 6 is equal to the tibiofemoral flexion angular velocity ($^{{\text{T}}} {{\varvec{\omega}}}^{{\text{F}}}$) multiplied by radius of the femoral condyle ($^{{\text{T}}}{\varvec{r}}^{{\text{OC}}}$), and is a measure of the contribution of rotation of the tibiofemoral joint to the slip velocity at the tibiofemoral contact center. If the magnitude of the relative velocity is equal to the linear velocity of the center of the femoral condyle, the velocity of the tibiofemoral contact center would be zero, and the femoral condyle would then roll on the tibial bearing. *HS* heel‐strike, *CTO* contralateral toe‐off, *CHS* contralateral heel‐strike, *TO* toe‐off

Why is tibiofemoral slip velocity linearly related to tibiofemoral (knee) flexion angular velocity (Fig. 5)? For each activity and each TKA design, the time history of tibiofemoral slip velocity mirrored the time history of tibiofemoral flexion angular velocity (Fig. 3). Fig. 4 shows that peak-to-peak slip velocities in the anterior-posterior direction were more than 1 order of magnitude larger than those measured in the medial-lateral direction, reinforcing that the relative movements of the femur and tibia are confined mainly to the sagittal plane. Assuming to a first approximation sagittal-plane motion of the knee joint, the velocity of the tibiofemoral contact point fixed on the femur, point C, may be expressed as (see Fig. 6):

5 $$^{{\text{T}}} {\varvec{v}}^{{\text{C}}} = ^{{\text{T}}} {\varvec{v}}^{{\text{O}}} + ^{{\text{T}}} {\varvec{\omega}}^{{\text{F}}} \times ^{{\text{T}}} {\varvec{r}}^{{{\text{OC}}}}$$

where the individual terms appearing in equation (5) are defined in Fig. 6. In Fig. 7, we plot the time histories of the anterior-posterior slip velocities (${{\text{T}}} {{\varvec{v}}}^{{\text{C}}}$, the term on the left-hand side of equation (5)), anterior-posterior velocities of the centers of the medial and lateral femoral condyles ($^{{\text{T}}} {{\varvec{v}}}^{{\text{O}}}$, the first term on the right-hand side), and the corresponding velocities of the medial and lateral contact points relative to each condylar center ($^{{\text{T}}} {{\varvec{\omega}}}^{{\text{F}}} \times ^{{\text{T}}}{\varvec{r}}^{{\text{OC}}}$, the second term on the right-hand side). For all 3 TKA designs and all activities, the magnitude of $^{{\text{T}}} {{\varvec{v}}}^{{\text{O}}}$ was much smaller than the magnitude of $^{{\text{T}}} {{\varvec{\omega}}}^{{\text{F}}} \times ^{{\text{T}}}{\varvec{r}}^{{\text{OC}}}$ (Fig. 7, compare second and third rows for the medial compartment and the lateral compartment), indicating that the velocity of the contact point ($^{{\text{T}}} {{\varvec{v}}}^{{\text{C}}}$) was dominated by the velocity of the contact point relative to the condylar center ($^{{\text{T}}} {{\varvec{\omega}}}^{{\text{F}}} \times ^{{\text{T}}}{\varvec{r}}^{{\text{OC}}}$) in both the medial and lateral compartments. Noting that the magnitude of the position vector from the condylar center to the contact point, $^{{\text{T}}}{\varvec{r}}^{{\text{OC}}}$, represents the radius of the femoral condyle at each instant, which varied little during each activity, it follows that slip velocity, $^{{\text{T}}} {{\varvec{v}}}^{{\text{C}}}$, was directly proportional to the flexion angular velocity, $^{{\text{T}}} {{\varvec{\omega}}}^{{\text{F}}}$. This then explains why slip velocity is linearly related to the tibiofemoral (knee) flexion angular velocity across all TKA designs and all activities (Fig. 5).

Our measurements of the relative movements of the femur and tibia may be used to deduce whether the femoral condyles roll, slide, or slip on the tibial bearing during each activity. Sliding and slipping of the femoral component on the tibial bearing are two types of movement caused by the translation and rotation of the femoral component, respectively. Sliding may be quantified by the velocity of the condylar center, $^{{\text{T}}} {{\varvec{v}}}^{{\text{O}}}$, whereas slipping may be quantified by the angular velocity of the femur, $^{{\text{T}}} {{\varvec{\omega}}}^{{\text{F}}}$, or by the velocity of the contact point relative to the condylar center, $^{{\text{T}}} {{\varvec{\omega}}}^{{\text{F}}} \times ^{{\text{T}}}{\varvec{r}}^{{\text{OC}}}$. The movement observed at the counterface involves a combination of sliding and slipping. Rolling, on the other hand, is a special case where the effects of sliding and slipping negate each other, resulting in zero velocity of the contact point, i.e., $^{{\text{T}}} {{\varvec{v}}}^{{\text{C}}}=0$. Therefore, the kinematic conditions at the counterface may be determined by the following rules, where boldface quantities denote vectors and | | represents the magnitude of a vector:

- If $$\left|{^{{\text{T}}} {{\varvec{v}}}^{{\text{C}}} }\right|=0$$, rolling;
- If $\left|{^{{\text{T}}} {{\varvec{\omega}}}^{{\text{F}}}}\right|=0$ and $\left|{^{{\text{T}}} {{\varvec{v}}}^{{\text{O}}}}\right|>0$, pure sliding;
- If $\left|{^{{\text{T}}} {{\varvec{\omega}}}^{{\text{F}}}}\right|>0$ and $\left|{^{{\text{T}}} {{\varvec{v}}}^{{\text{O}}}}\right|=0$, pure slipping;
- Otherwise, a combination of sliding and slipping exists.

The amount of sliding and slipping may be determined by comparing the magnitudes of $^{{\text{T}}} {{\varvec{v}}}^{{\text{O}}}$ and $^{{\text{T}}} {{\varvec{\omega}}}^{{\text{F}}} \times ^{{\text{T}}}{\varvec{r}}^{{\text{OC}}}$. Applying these rules to the results of Fig. 7 we conclude that slipping is the predominant behavior present during the stance phase of each activity when the limb is weightbearing, and that a combination of sliding and slipping presides during the swing phase when the limb is not in contact with the ground.

These findings have relevance for the wear of polyethylene bearing surfaces in prosthetic knees. Blunn *et al.* [9] found that a spherical-ended metal femoral component rolling on a flat polyethylene tibial plateau produced minimal surface damage and wear, whereas sliding (and presumably slipping) motions yielded severe surface and subsurface cracking resulting in high wear. In all 5 activities of daily living investigated in the present study, slipping and sliding were the dominant forms of relative motion at the counterface, with rolling observed only minimally. This finding emphasizes the importance of incorporating realistic *in vivo* measurements of slip velocity in joint simulator experiments and computational models of wear, particularly during weightbearing when peak compressive loads are typically between 2 and 4 times body weight [13, 17, 27].

Based on their results of *in vitro* experimental simulations of TKA wear, Blunn *et al.* [9] concluded that low-conformity components are likely to be more susceptible to anterior-posterior sliding, and hence wear, compared to more-conforming components that limit sliding motions and reduce contact stresses. The results of Fig. 4 imply that this may not necessarily be the case. We found that peak anterior-posterior slip velocities across all 5 activities in the conforming MS design were comparable to those generated in the non-conforming PS and CR designs. Furthermore, peak medial-lateral slip velocities in the highly conforming medial compartment of MS were much higher than those generated in PS and CR (Fig. 4, first row, compare MS with PS and CR for level walking). Peak slip velocities in the medial compartment of MS were comparable if not higher than those measured for PS and CR because the MS design functioned primarily as a hinge, where the medial sphere of the femoral component slipped inside its highly conforming medial tibial socket. Slipping was the predominant motion observed for MS because this design limited the translations of the femur relative to the tibia, and hence kept sliding to a minimum. So, even though the motion path of the tibiofemoral contact point was constrained to a much smaller distance on the tibia in MS (Fig. S2, compare MS to PS and CR in bottom row), the magnitude of slip velocity was not reduced by the highly conforming medial compartment. These results are consistent with the view that peak slip velocity, total slip distance, and therefore potential wear, are all more sensitive to activity type than the geometry of the knee implant.

One of the main findings of this study is that tibiofemoral slip velocity may be estimated from a measurement of tibiofemoral (knee) flexion angular velocity. The slope of the linear relationship between slip velocity (in mm/s) and flexion angular velocity (in rad/s) across all TKA designs and all activities was 26.9 mm for the medial compartment and 26.1 mm for the lateral compartment (Fig. 5, All Activities). These values are practically the same as the mean radii of the medial and lateral femoral condyles (27.0 mm for both) across all TKA designs. Because the velocity of each condylar center was much smaller than that of the contact point (slip velocity) (Fig. 7), the slope of each line drawn in Fig. 5 should reflect the radius of the medial or lateral femoral condyle. Thus, as illustrated in Fig. 5, for any activity, irrespective of TKA design, tibiofemoral slip velocity may be estimated by multiplying the measured flexion angular velocity (in rad/s) by the radius of each femoral condyle, where the angular velocity can also be measured using video motion capture with skin-mounted markers or inertial measurement units.

Our measurements of the trajectories of the tibiofemoral contact centers and slip velocities in the medial and lateral compartments of the PS, CR and MS designs may be used, along with published data on joint loading [13], as input data in experimental and computational studies aimed at evaluating TKA wear. Bragdon *et al.* [28] found that physiological motion pathways produce very different wear rates and morphology of the wear surface than simple unidirectional reciprocating pathways. Turell *et al.* [29] showed that multidirectional or cross-shear motion at the counterface has a significant impact on the wear rate of polyethylene components used in total hip replacements. Fig. 4 illustrates the extent to which multidirectional motion paths are generated during each cycle of an activity of daily living, and the time histories of these pathways (available in the Supplemental Material) may be used to drive robotic joint simulators in *in vitro* experiments designed to more accurately reproduce variations in sliding direction responsible for high rates of volumetric wear [5].

**Fig. 8:**
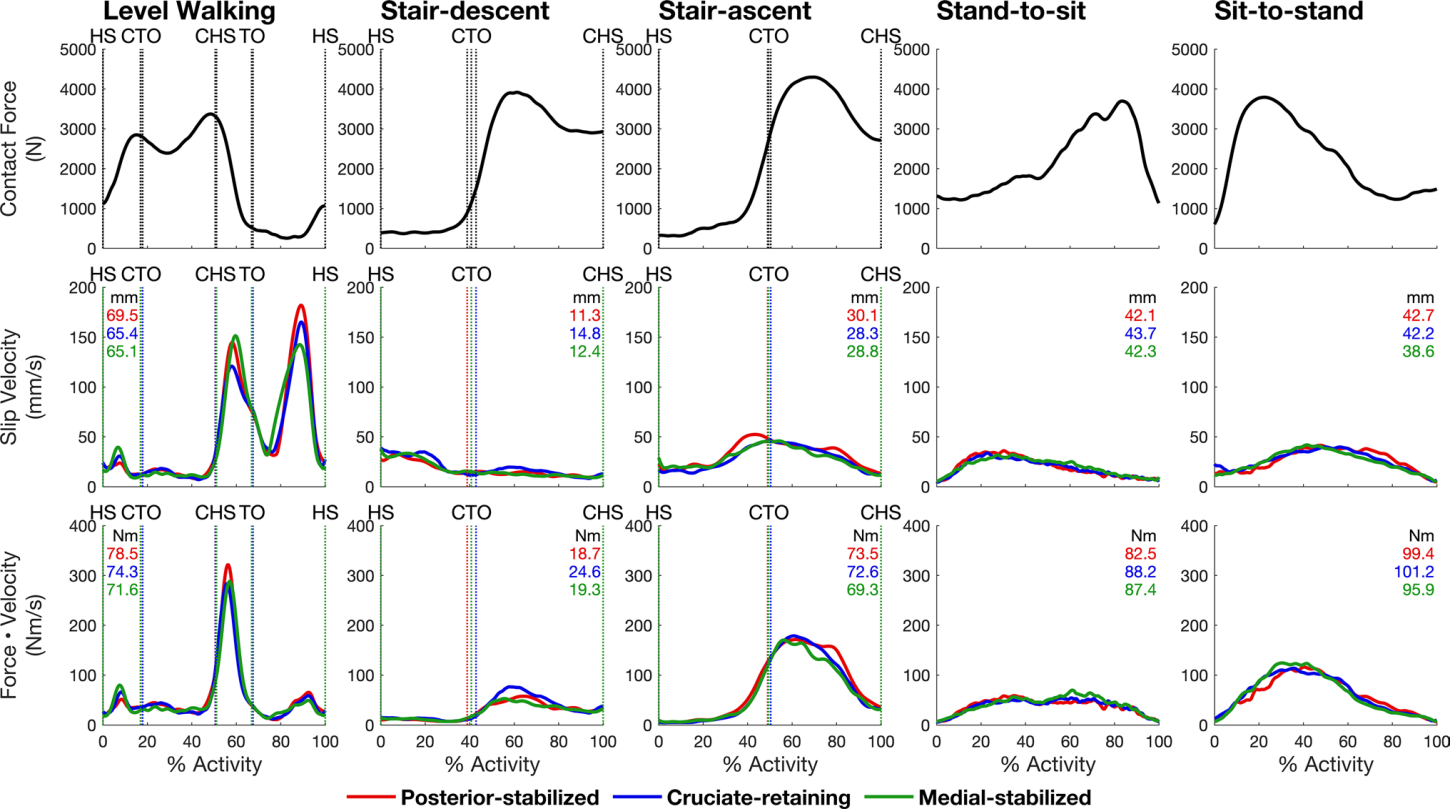
Tibiofemoral contact force (top row), slip velocity averaged across the medial and lateral compartments (second row), and force-velocity factor (i.e., wear rate, bottom row) for the 5 activities tested. Data were resampled at 201 equally spaced time points from the beginning to the end of each activity. Tibiofemoral contact force was obtained from Bergmann *et al.* [34] (“High100” force, loads at high level in subject with 100 kg body weight), and the magnitude of slip velocity was found by averaging the data shown in Fig. 2 for the medial and lateral compartments of the tibiofemoral joint. Step-down and step-up activities in the present study approximated the stair-descent and stair-ascent activities reported by Bergmann *et al.* [34]. Knee flexion angles measured in the two studies were matched to ensure the tibiofemoral contact forces used were appropriate. Flexion angles were used to select the region of contact forces matching our activities. The numbers in red, blue, and green represent the area calculated under each curve plotted for the slip velocity and the force-velocity factor and correspond to the posterior-stabilized, cruciate-retaining and medial-stabilized designs, respectively. The areas under the curves for the slip velocity and the force-velocity factor represent slip distance and TKA wear, respectively. The vertical dotted lines mark key events during each activity cycle: *HS* heel‐strike, *CTO* contralateral toe‐off, *CHS* contralateral heel‐strike, *TO* toe‐off

Our kinematic data also may be used as inputs to computational models used to predict wear rate *in vivo*. Knowlton *et al.* [30] used measurements of sliding distance along the contact path obtained from gait analysis experiments together with published estimates of joint load as inputs to Archard’s Law to predict volumetric wear rates in prosthetic knees. We performed a similar analysis to illustrate the effect of TKA design and activity type on wear rate using Archard’s Law for mild wear [4]. Fig. 8 shows that wear rate (estimated by the force-velocity factor) and the amount of wear (estimated by the area under the force-velocity factor curve), like slip velocity and total slip distance, are invariant to TKA design but dependent on activity type. Interestingly, the third peak in slip velocity for level walking did not produce a substantial wear rate due to the relatively low contact force present during the swing phase. Whereas peak slip velocity and total slip distance over one full cycle were significantly higher for level walking than for all the other activities, wear was highest for sit-to-stand (mean ~ 98 Nm), moderately lower for stand-to-sit, level walking, and stair ascent (mean ~ 79 Nm), and substantially lower for stair descent (mean ~ 21 Nm). Level walking exhibited ~ 20% less wear compared to sit-to-stand; however, it is the most frequently performed activity in daily life [31]. We suggest therefore that joint simulator experiments designed to measure TKA component wear prioritize level walking for *in vitro* testing.

Previous estimates of tibiofemoral slip velocity during TKA gait vary considerably. Seedhom *et al.* [8] estimated a peak slip velocity of ~ 275 mm/s that occurred during the early stance phase of gait. Johnson *et al.* [6] reported two prominent peaks in tibiofemoral slip velocity, one measuring ~ 150 mm/s at toe-off and the other reaching ~ 200 mm/s during terminal swing. These authors also found peak slip velocity to be higher in the medial compartment than the lateral compartment, which is opposite to our findings for level walking, but consistent with the results obtained for the other activities (Table 2 and Table 3). Andriacchi *et al.* [32] reported a much higher peak slip velocity of nearly 500 mm/s in the lateral compartment of a TKA prosthesis during terminal swing. Schwenke *et al* [10]*.* found two peaks in slip velocity during early and late stance, both similar in magnitude and ranging from 150 to 175 mm/s. Guan *et al.* [22] measured slip velocities in the medial and lateral compartments of a PS design during overground and treadmill gait and reported peaks of ~ 150 mm/s during late stance and late swing in both conditions. Dumas *et al.* [18] also found two peaks in slip velocity during late stance and late swing, each measuring ~ 200 mm/s in both the medial and lateral compartments. Our measurements of peak slip velocity for TKA gait agree closely with those given by Guan *et al.* [22] and compare reasonably well with estimates reported by Johnson *et al.* [6], Schwenke *et al.* [10] and Dumas *et al.* [18]. We found that tibiofemoral slip velocity is highest during terminal stance and mid-swing for both the medial and lateral compartments across all TKA designs (range: 117 – 185 mm/s) (Fig. 2). Our results for the step-up are also in good agreement with those of Hamilton *et al.* [5] who used single-plane X-ray fluoroscopy to measure slip velocity during stair ascent. They found peak slip velocities of ~ 40 mm/s in the lateral compartment of a TKA knee, consistent with our estimates of mean peak slip velocity in the lateral compartment of all 3 TKA designs (range: 42–47 mm/s) (Fig. 2).

There are limitations associated with the estimates of tibiofemoral slip velocity reported here. First, patient testing occurred 6 months after TKA surgery, and it is possible that TKA tibiofemoral kinematics change over time. Mizner *et al.* [33] reported that quadriceps strength, knee joint range of motion, and self-reported function all stabilized by the third month after TKA surgery, suggesting that other metrics such as tibiofemoral flexion angular velocity, which was the main determinant of slip velocity, may also have stabilized by this time. Second, we calculated slip velocity by evaluating the position and velocity of the closest point of each femoral condyle relative to the surface of the tibial bearing. This method cannot verify whether articular contact between the femoral component and tibial bearing was always present. Some previous studies have reported condylar lift-off during gait and other activities (e.g., Dennis *et al.* [15]). If, for example, condylar lift-off occurred during the swing phase of level walking, wear rate would be zero because joint load then would be zero. Thus, the results of Fig. 8 are likely to overestimate wear rate if the femoral component and tibial bearing were to separate during certain periods of an activity. Third, the indicative estimates of wear rate given in Fig. 8 are based on Archard’s Law for mild wear and do not consider transverse crossing motions which are known to increase wear rate significantly. However, Knowlton *et al.* [30] showed that Archard’s equation for mild wear explained 57% of the variability in the total wear measured from TKA prostheses when patient-specific gait inputs were used.

In summary, we found that the pattern of tibiofemoral slip velocity was similar for all TKA designs within each activity but markedly different across the various activities tested, with the magnitude of peak slip velocity being highest in level walking. The pattern of tibiofemoral slip velocity in both the medial and lateral compartments closely resembled the pattern of knee joint angular velocity, with a strong linear relationship observed between slip velocity and flexion angular velocity (*r* = 0.81–0.97). These results may be used as inputs in joint simulator experiments and computational models used to estimate TKA component wear.

## Supplementary Information

Below is the link to the electronic supplementary material.

Supplementary file1 (XLSX 547 KB)

Supplementary file2 (DOCX 992 KB)
