# Supplementary material for: Tibiofemoral Slip Velocity in Total Knee Arthroplasty is Design-Invariant but Activity-Dependent
Source: Ann Biomed Eng. 2024 Mar 26;52(6):1779–94. doi: 10.1007/s10439-024-03490-4 (PMC11560988; doi:10.1007/s10439-024-03490-4)
Supplement: Supplementary file 2 — Supplementary file2 (DOCX 992 KB) [file 10439_2024_3490_MOESM2_ESM.docx]

**SUPPLEMENTAL MATERIAL**

**TIBIOFEMORAL SLIP VELOCITY IN TOTAL KNEE ARTHROPLASTY IS DESIGN-INVARIANT BUT ACTIVITY-DEPENDENT**

Shanyuanye Guan^1^, Raphael Dumas^2^, Marcus G Pandy^1^

^1^Department of Mechanical Engineering, University of Melbourne, Parkville, Victoria, Australia

^2^University of Lyon, University Gustave Eiffel, Univ Claude Bernard Lyon 1, LBMC UMR T_9406, F-69622 Lyon, France


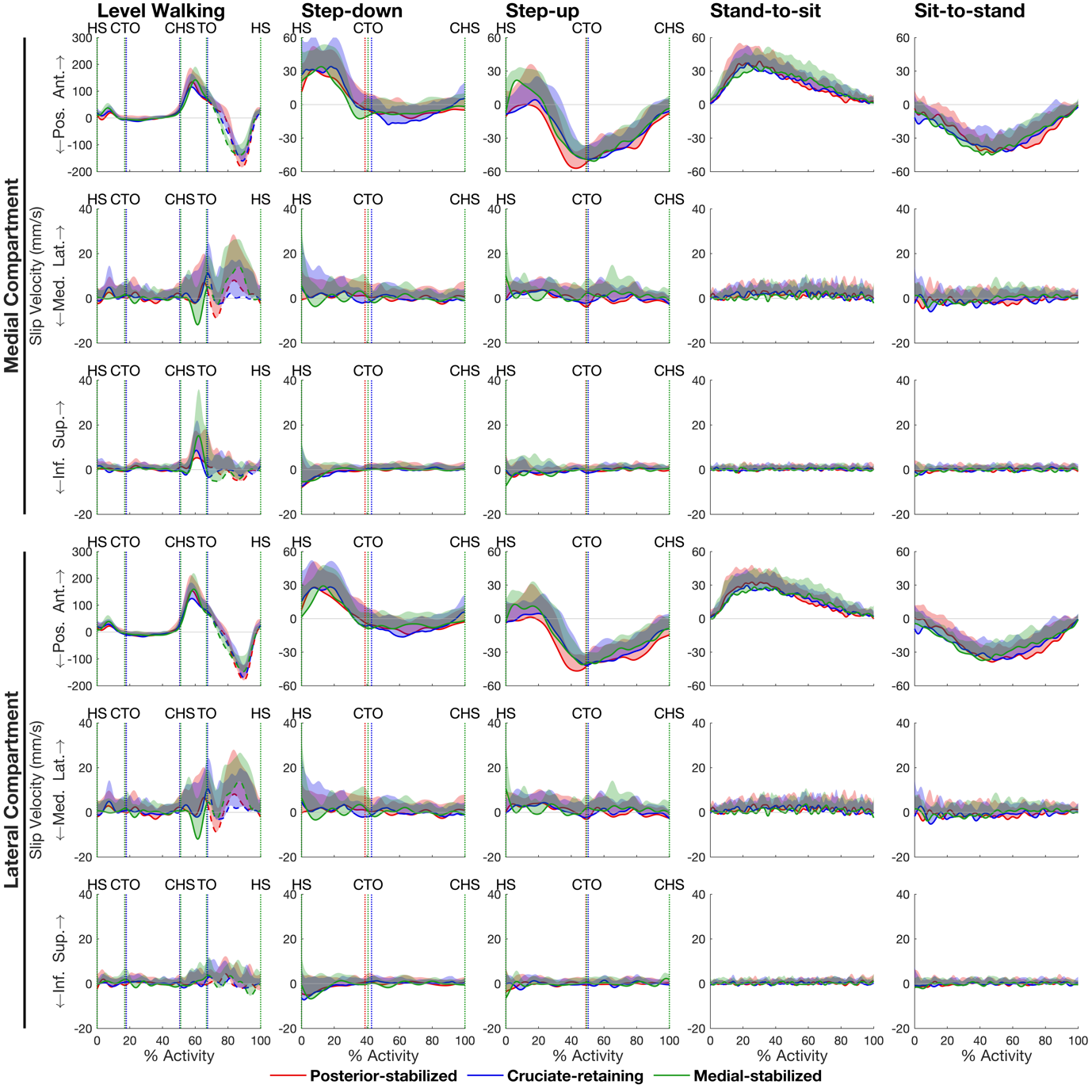


**Supplementary Figure S1.** Time histories of slip velocities in the anterior-posterior, medial-lateral, and inferior-superior directions for the medial compartment (top three rows) and the lateral compartment (bottom three rows) of the tibiofemoral joint for the three TKA designs and five activities tested. Slip velocities were calculated with respect to the reference frame fixed on the tibial bearing. Results **were obtained at 201 equally spaced time points between the beginning and end of each activity.** The solid and dashed lines represent the mean sliding velocity across all participants in each group while the shaded regions represent 1 standard deviation from the mean. HS, heel‐strike; CTO, contralateral toe‐off; CHS, contralateral heel‐strike; TO, toe‐off; Pos., posterior; Ant., anterior; Med., medial; Lat., lateral; Inf., inferior; Sup., superior.


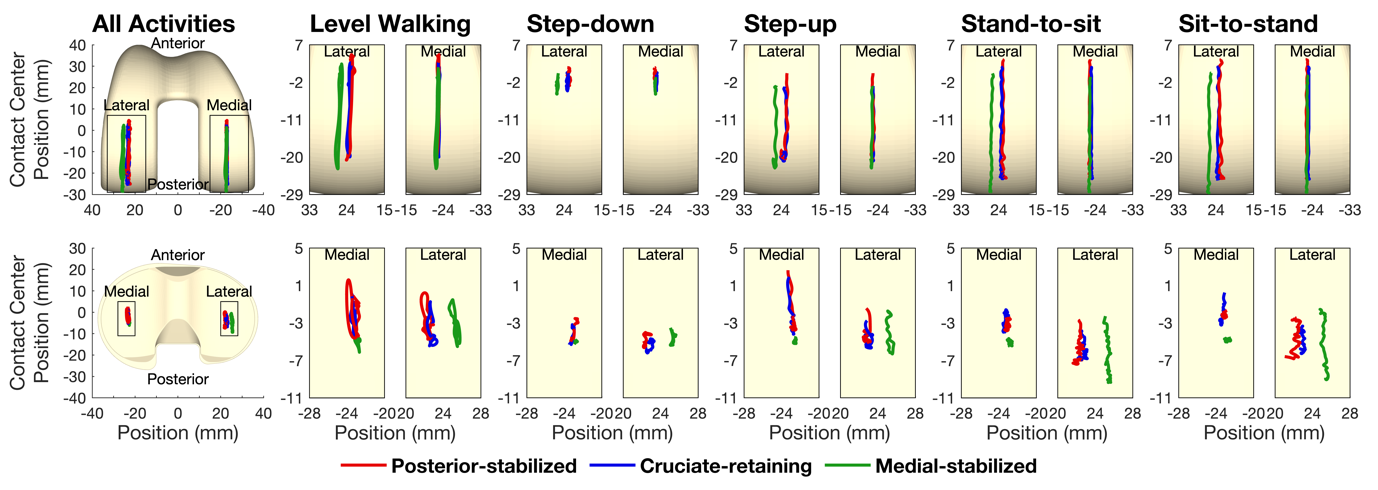


**Supplementary Figure S2.** Mean locations of the contact center in the medial and lateral compartments of the tibiofemoral joint projected onto the condyles of the femoral component (top row) and the tibial bearing (bottom row) for the three TKA designs and five activities. The insets (columns 2-6) show magnified views of the trajectories of the tibiofemoral contact centers on the condyles of the femur and tibial bearing for each activity.

**Supplementary Table S1.** The distribution of prosthesis sizes and radii of the femoral condyles.

|  | **Posterior-stabilized** | |  | **Cruciate-retaining** | |  | **Medial-stabilized** | |
| --- | --- | --- | --- | --- | --- | --- | --- | --- |
| Size | Number of Participants | Radius  (mm) |  | Number of Participants | Radius  (mm) |  | Number of Participants | Radius  (mm) |
| 2 |  |  |  | 1 | 22.09 |  | 1 | 25.00 |
| 3 | 3 | 23.85 |  | 5 | 23.85 |  | 3 | 25.00 |
| 4 | 5 | 25.51 |  | 8 | 25.51 |  | 8 | 30.00 |
| 5 | 8 | 26.09 |  | 3 | 26.09 |  | 8 | 30.00 |
| 6 | 6 | 27.02 |  | 8 | 27.02 |  | 5 | 30.00 |
| 7 | 1 | 27.32 |  | 1 | 27.32 |  | 1 | 30.00 |
|  | **Sum 23** | **Mean 25.97** |  | **Sum 26** | **Mean 25.66** |  | **Sum 26** | **Mean 29.23** |
